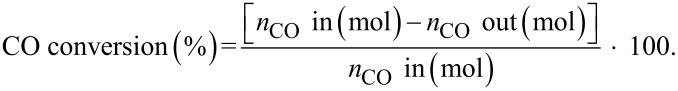# Nanocrystalline ZrO_2_ and Pt-doped ZrO_2_ catalysts for low-temperature CO oxidation

**DOI:** 10.3762/bjnano.8.29

**Published:** 2017-01-26

**Authors:** Amit Singhania, Shipra Mital Gupta

**Affiliations:** 1 Department of Chemical Engineering, Indian Institute of Technology Delhi, Hauz Khas, New Delhi 110016, India; 2 University School of Basic and Applied Sciences, Guru Gobind Singh Indraprastha University, Sector 16-C, Dwarka, New Delhi 110078, India

**Keywords:** CO oxidation, nanomaterials, platinum, solution combustion method, zirconia

## Abstract

Zirconia (ZrO_2_) nanoparticles were synthesized by solution combustion using urea as an organic fuel. Brunauer–Emmett–Teller (BET), X-ray diffraction (XRD), thermal gravimetric analysis (TGA), transmission electron microscopy (TEM), UV–vis and Fourier transform infrared (FTIR) measurements were performed in order to characterize the catalyst. The calculated crystallite size of ZrO_2,_ calculated with the help of the Scherrer equation, was around 30.3 nm. The synthesized ZrO_2_ was scrutinized regarding its role as catalyst in the oxidation of carbon monoxide (CO). It showed 100% CO conversion at 240 °C, which is the highest conversion rate reported for ZrO_2_ in literature to date. It is found that through solution combustion, Pt^2+^ ions replace Zr^4+^ ions in the ZrO_2_ lattice and because of this, oxygen vacancies are formed due to charge imbalance and lattice distortion in ZrO_2_. 1% Pt was doped into ZrO_2_ and yielded excellent CO oxidation. The working temperature was lowered by 150 °C in comparison to pure ZrO_2_. Further, it is highly stable for the CO reaction (time-on-stream ≈ 40 h). This is because of a synergic effect between Pt and Zr components, which results in an increase of the oxygen mobility and oxygen vacancies and improves the activity and stability of the catalyst. The effects of gas hourly space velocity (GHSV) and initial CO concentration on the CO oxidation over Pt(1%)-ZrO_2_ were studied.

## Introduction

Nanomaterials received a lot of attention from researchers because of their different and interesting optical, electrical, thermal, catalytic and magnetic properties that differ from those of the bulk materials [1–2]. Zirconium oxide (ZrO_2_) is an important and extensively studied ceramic material and is widely used in the industry. It is a wide band gap semiconductor (5–7 eV) and is known for its unique mechanical, electrical, thermal, catalytic and optical capabilities [3–4]. ZrO_2_ finds a range of applications in different fields such as catalyst/support, as biomaterial [5], as refractory metal [6], in thermal barrier coating [7], gas sensors [8], in solid oxide fuel cells [9], in ceramic production, insulation and abrasives.

Carbon monoxide (CO) is considered a major pollutant and it causes serious health problems. It is important to control CO released from natural sources and anthropogenic activities. The catalytic CO oxidation is a very well established and exploited process. So far, noble metals such as Pt, Pd, Rh and Au dominated as catalysts for CO oxidation [10–12]. Various supports such as Al_2_O_3_, TiO_2_, SiO_2_, CeO_2_, Fe_2_O_3_ and carbon nanotubes (CNTs) have also been used for CO oxidation by different researchers [13–16]. The addition of precious metals such as Pd, Pt and Rh increased the reactivity of the support by increasing its oxygen mobility and number of oxygen vacancies (the source of oxygen in CO oxidation) [10–12].

In recent years, ZrO_2_ has been used as catalyst/support because of its high activity and thermal stability. The advantages also include its inertness under acidic reaction environments [17]. Also, it is reported as a better catalyst and support than many other materials, such as Al_2_O_3_, TiO_2_ and SiO_2_ [18]. It has been used by different researchers in several important catalytic reactions such as autothermal reforming of ethanol [19], in solid oxide fuel cells [9] and hydrogenation reactions [20]. The addition of Pt to ZrO_2_ can increase the oxygen vacancies and oxygen storage capacity, which play a major role in lowering the CO oxidation temperature. Also, this addition results in an increase in surface area and stability of the ZrO_2_ material [17]. Hence, ZrO_2_ and the combination of Pt and ZrO_2_ are promising catalysts for the oxidation of CO.

Different researchers have employed various methods for the preparation of ZrO_2_ nanoparticles such as sol–gel [21], precipitation [22], combustion [23], hydrothermal synthesis [24], solvothermal synthesis [25], reverse micelles [26], chemical vapor synthesis [27], aerosol pyrolysis [28], and sonochemical synthesis [29]. Dongare et al. [30] described the synthesis of ZrO_2_ by a sol–gel method. Tartaj et al. [31] prepared nanospherical ZrO_2_ particles using a microemulsion-mediated process. Among these preparation methods, solution combustion method is useful because it is an easy process, requiring less time and producing high purity products. It is therefore quite promising for industrial applications. The above method involves combustion of an aqueous solution of metal nitrates and a fuel (glycine [32], citric acid [33], sucrose [4] and hexamethylenetetramine [34]). The combustion reaction is an exothermic redox reaction that takes place between metal nitrate and the fuel. Nanomaterials are produced by the disintegration of reaction precursors resulting from a quick expulsion of gases. In this method, fixed proportions of an oxidizer and the fuel are used and this results in a large amount of heat energy being released.

In this paper, we have described the use of solution combustion using urea as an organic fuel and metal nitrates as oxidizer, to produce high-purity ZrO_2_ nanoparticles. This method was used with intent to produce stable nanoparticles with high catalytic activity. Also, Pt was doped into the ZrO_2_ lattice by solution combustion to scrutinize the ZrO_2_ material as support. This paper also reports the characterization of the prepared catalysts by BET, XRD, TGA, TEM, UV–vis and FTIR measurements.

## Results and Discussion

### Materials characterization

The specific surface area, pore volume and pore diameter of ZrO_2_ and Pt(1%)-ZrO_2_ were obtained carrying out BET nitrogen adsorption measurements. Table 1 shows the BET surface area of 34.5 m^2^·g^−1^ and 40.1 m^2^·g^−1^ for ZrO_2_ and Pt(1%)-ZrO_2_. The pore volume and pore diameter of ZrO_2_ are found to be 0.043 × 10^−6^ m^3^ g^−1^ and 9.4 nm, respectively. The incorporation of Pt into ZrO_2_ lattice is expected to cause lattice distortion that ultimately results in an increase of the surface area. The decrease in crystallite size and lattice constants further confirmed the lattice distortion (Table 1). The particle size *D*_p_ of the ZrO_2_ nanoparticles could be calculated using specific surface area by the following equation:


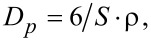


where *S* is the specific surface area (m^2^·g^−1^) and ρ is the density of the sample (5.33 × 10^6^ g m^−3^) [4].

**Table 1:** Sample characterization by BET and XRD.

sample	surface area^a^ (m^2^·g^−1^_)_	pore volume^b^ × 10^−6^ (m^3^·g^−1^)	average pore diameter (nm)	crystallite size^c^ (nm)	lattice constants^d^ (Å)

ZrO_2_	34.5	0.043	9.4	30.3	5.1250
Pt(1%)-ZrO_2_^e^	40.1	0.059	9.2	26.7	5.1124

^a^BET surface area (3% error); ^b^total pore volume; ^c^calculated using Scherrer equation and the (111) reflection (2% error); ^d^calculated using Bragg’s Law and the (111) reflection; ^e^material composition was determined by ICP-AES.

**Figure 1:**
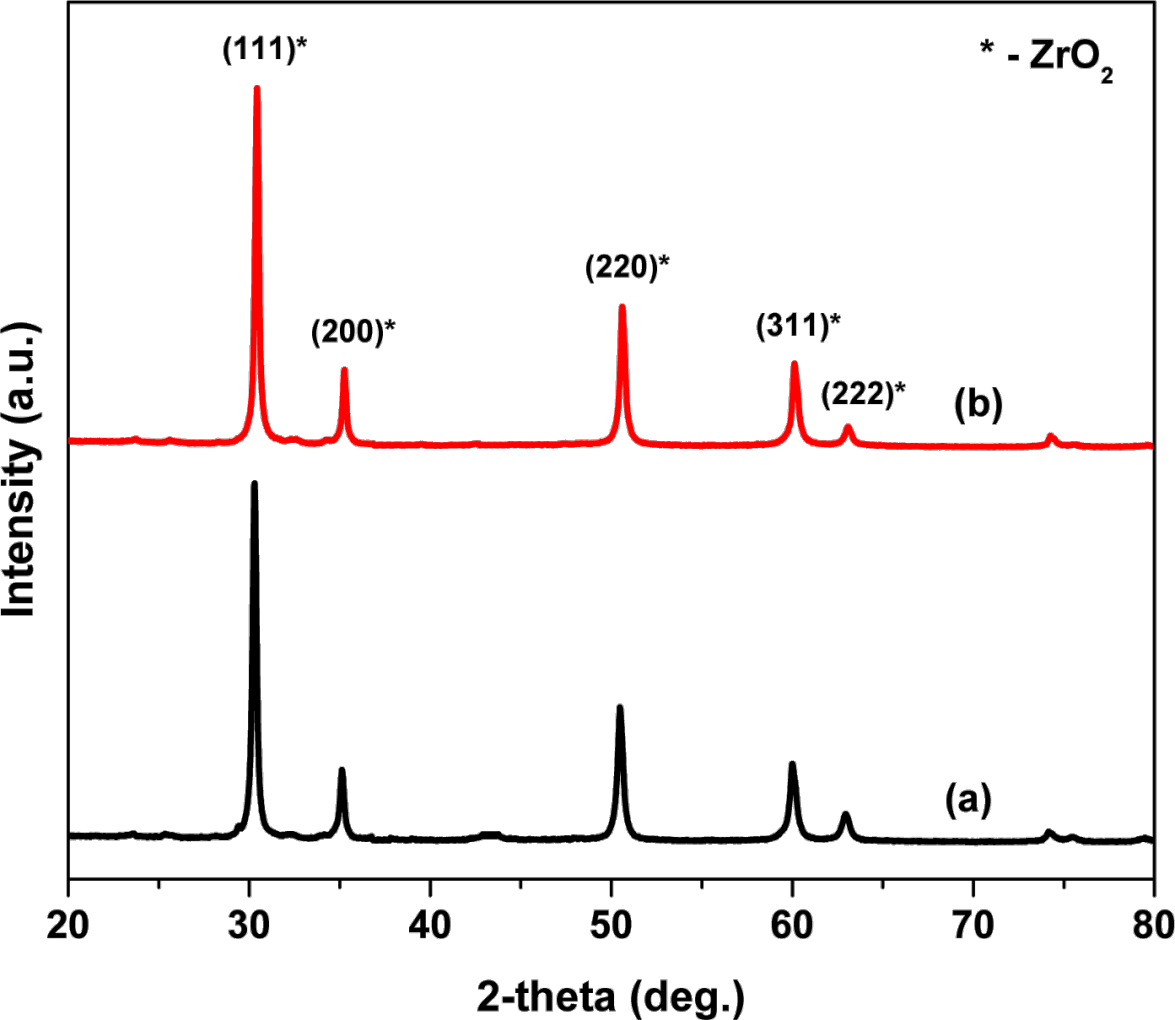
XRD patterns of (a) ZrO_2_, and (b) Pt(1%)-ZrO_2_.

Figure 1a shows the powder XRD pattern of synthesized ZrO_2_ and Figure 1b shows the powder XRD pattern of Pt(1%)-ZrO_2_. Both the powder XRD patterns showed the cubic phase of ZrO_2_. XRD patterns showed sharp peaks at 30.2°, 35.1°, 50.4°, 59.9° and 62.9° of to the (111), (200), (220), (311) and (222) planes, respectively (JCPDS card no. 27-0997). No peak of Pt at 2θ = 39.8° was observed in the XRD pattern of Pt(1%)-ZrO_2_. The crystallite size *D* of the ZrO_2_ nanoparticles was calculated with the help of the Scherrer equation:


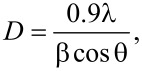


where β is the full-width at half-maximum (FWHM) in radians, λ is the wavelength of the used X-rays (Cu Kα radiation, λ = 1.5406 Å) and θ is the Bragg angle.

**Figure 2:**
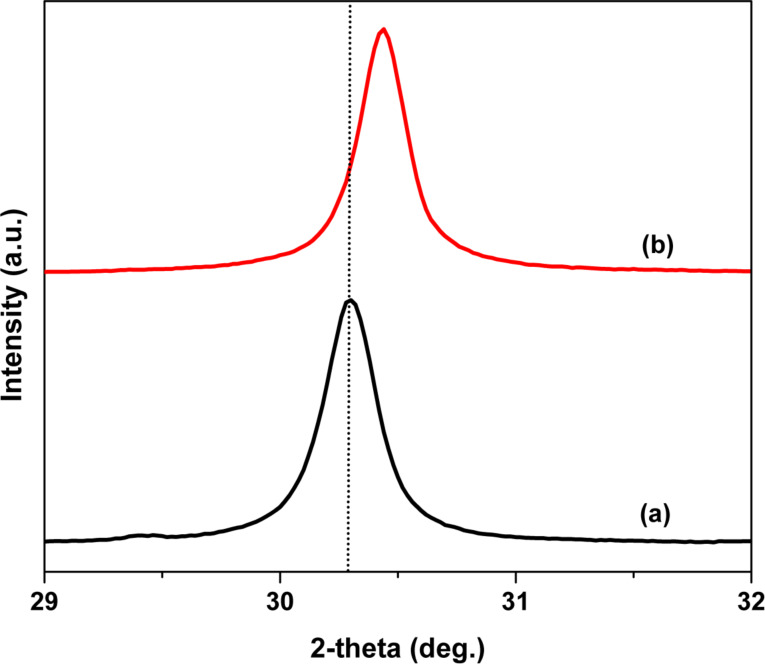
Expanded XRD region between 29° and 32° to show the peak shift between (a) ZrO_2_ and (b) Pt(1%)-ZrO_2_.

The calculated crystallite sizes of ZrO_2_ and Pt(1%)-ZrO_2_ were found to be 30.3 nm and 26.7 nm. The sharp ZrO_2_ peaks indicate the highly crystalline nature of the ZrO_2_ nanoparticles. Figure 2 shows an expanded region of the XRD pattern. The addition of Pt led to the shift of the diffraction peak to a higher angle (2θ) indicating the formation of a Pt–Zr solid solution.

The lattice parameters showed a decrease from 5.1250 Å to 5.1124 after the incorporation of Pt. The ionic radius of Pt^2+^ is 0.80 Å, which is smaller than the ionic radius of Zr^4+^ (0.86 Å). The decrease in lattice parameters indicates that the Pt^2+^ ions have dissolved into ZrO_2_ lattice during the synthesis. The solution of Pt^2+^ into ZrO_2_ the lattice created oxygen vacancies [35].

**Figure 3:**
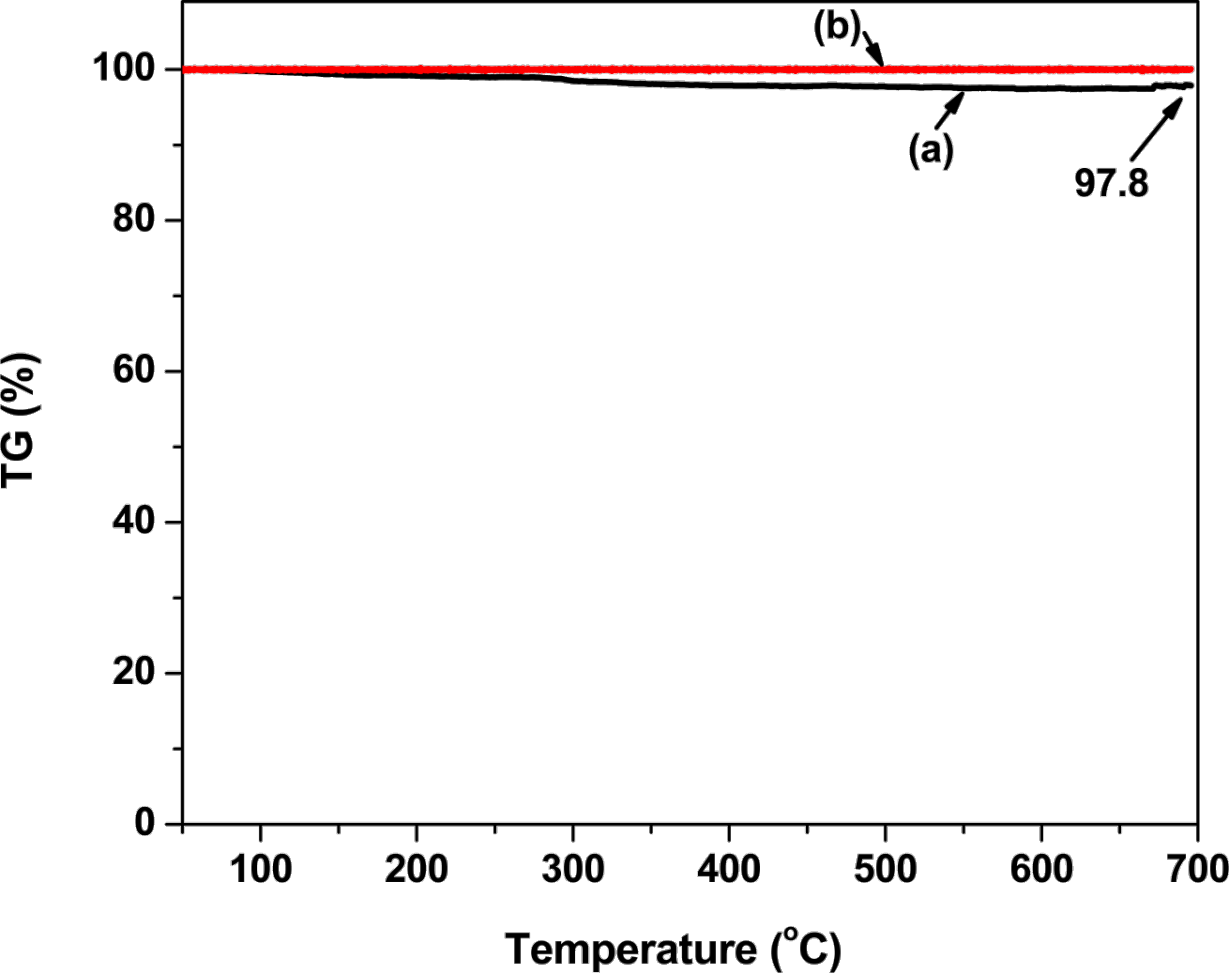
TGA of (a) ZrO_2_, and (b) Pt(1%)-ZrO_2_.

The thermal analysis of the prepared ZrO_2_ and Pt(1%)-ZrO_2_ nanoparticles is shown in Figure 3. The TGA curve shows an initial small weight loss between 50 and 100 °C, which may be due to evaporation of water adsorbed in the sample. After the initial weight loss, there was a small weight loss leading to a total loss of 2.2% in ZrO_2_ (slightly hydrated) indicating the excellent stability of ZrO_2_ nanoparticles under high temperatures (50–700 °C), which could prove to be an important factor in high-temperature industrial catalytic applications. The addition of Pt into ZrO_2_ showed an improvement in thermal stability as indicated by the TGA curve of Pt(1%)-ZrO_2_ in which there is no weight loss over the measured temperature range.

**Figure 4:**
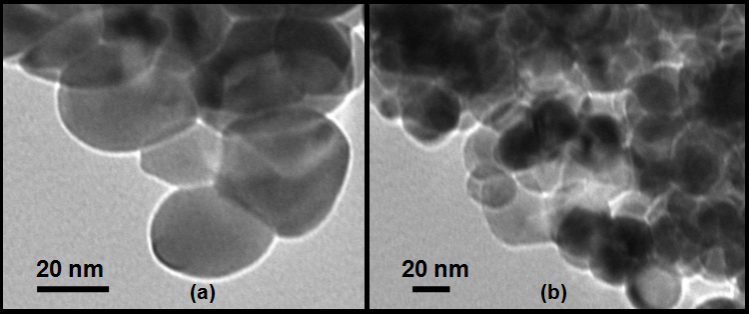
TEM micrograph of (a) ZrO_2_ and (b) Pt(1%)-ZrO_2_.

**Figure 5:**
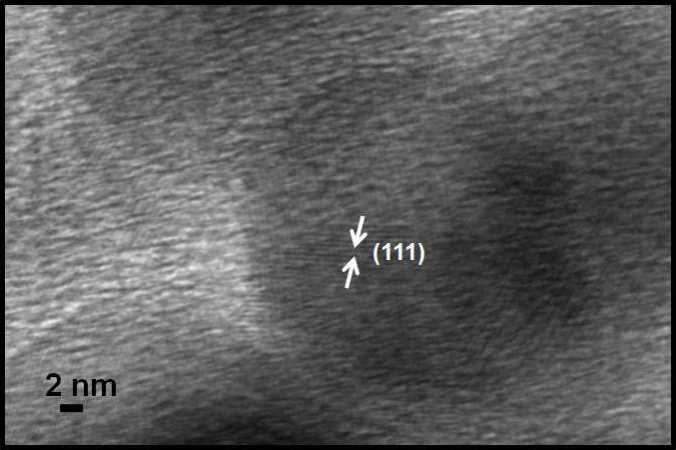
High-resolution TEM micrograph of Pt(1%)-ZrO_2_.

The TEM micrograph in Figure 4a shows ZrO_2_ nanoparticles of spherical shape and average size around 31.2 nm. Figure 4b shows Pt(1%)-ZrO_2_ particles with an average size of 28.1 nm. The incorporation of Pt into ZrO_2_ decreases the particle size. Figure 5 shows a high-resolution TEM micrograph of Pt(1%)-ZrO_2_. The *d*-spacing of zirconia is found to be 2.96 Å, which corresponds to the (111) plane spacing of ZrO_2_.

Table 2 shows the summary of ZrO_2_ and Pt(1%)-ZrO_2_ particle sizes using different analyses, namely, XRD, TEM and surface area measurements. The differently obtained values are in good agreement.

**Table 2:** Summary of ZrO_2_ and Pt(1%)-ZrO_2_ particle size from XRD, TEM and surface area measurements.

sample	XRD (crystallite size) (nm)	TEM (average particle size) (nm)	surface area measurement (particle size) (nm)

ZrO_2_	30.3	31.2	32.6
Pt(1%)-ZrO_2_	26.7	28.1	28.0

**Figure 6:**
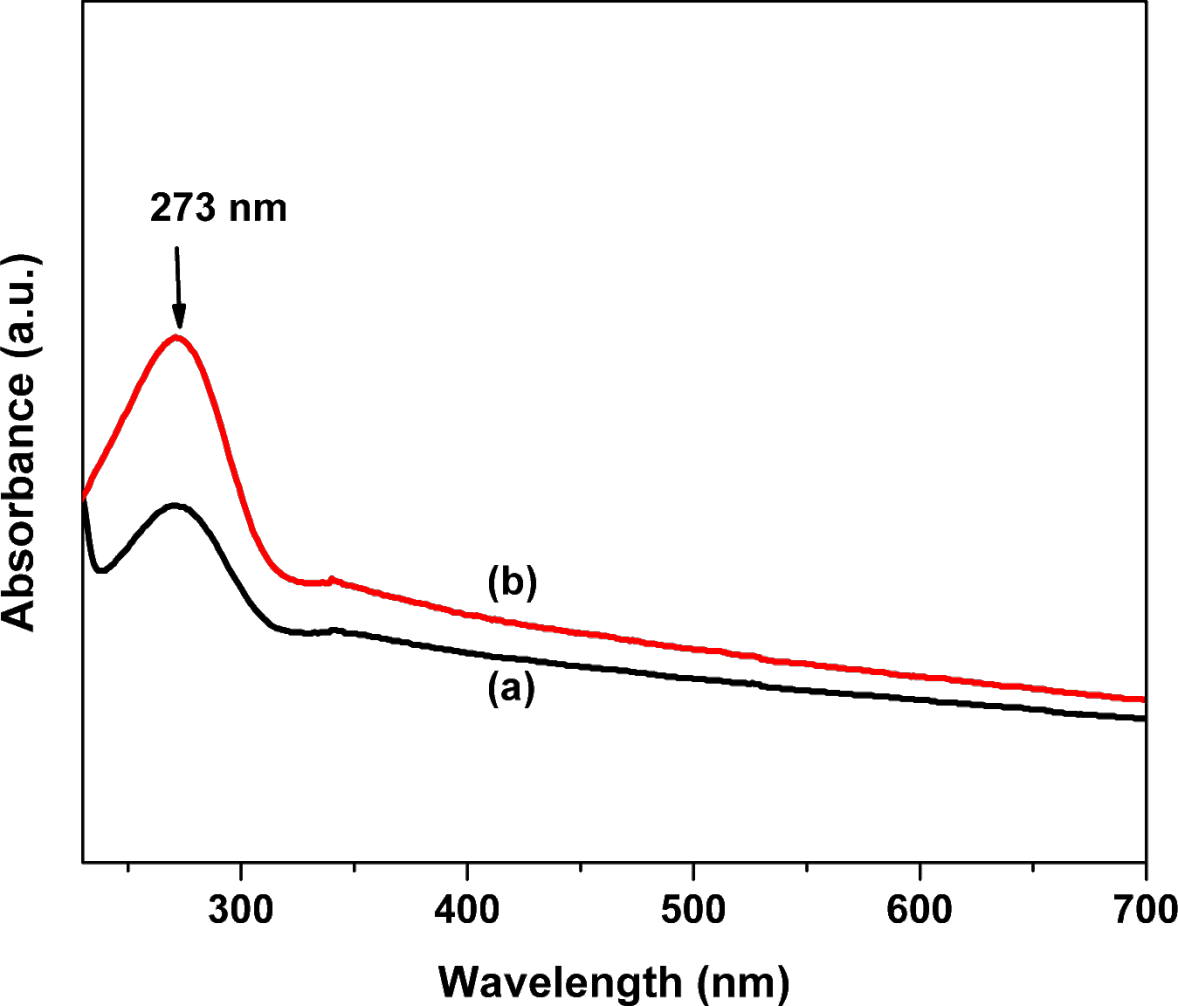
UV–vis spectra of (a) ZrO_2_ and (b) Pt(1%)-ZrO_2_.

UV–vis spectra of prepared ZrO_2_ and Pt(1%)-ZrO_2_ are shown in Figure 6. The sample for UV–vis analyses was obtained by dispersing ZrO_2_ samples in ethanol followed by sonication for about 30 min. The UV–vis spectra of both samples show a strong absorption peak at 273 nm, which agrees very well with data reported in literature [36]. The absorption peak in case of Pt(1%)-ZrO_2_ is higher than ZrO_2_ which could be beneficial for photocatalytic applications.

**Figure 7:**
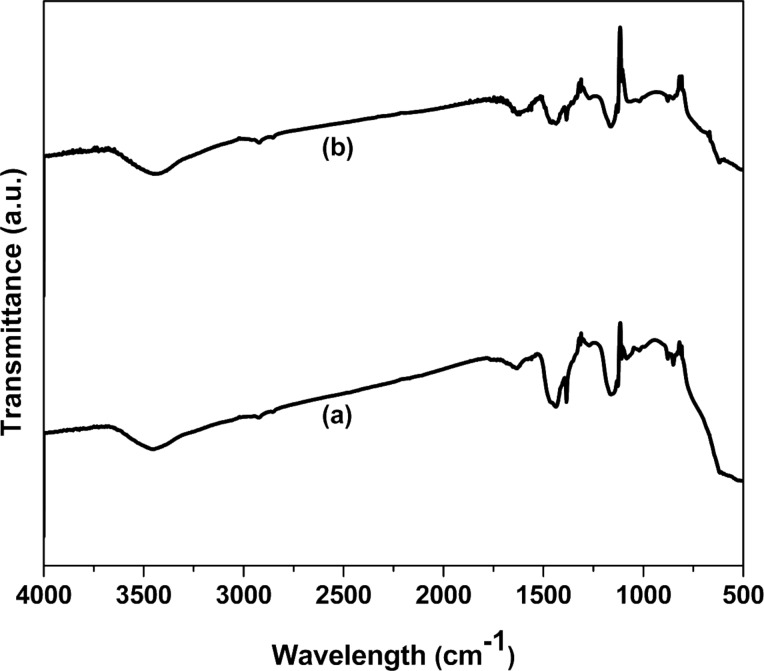
FTIR spectra of (a) ZrO_2_ and (b) Pt(1%)-ZrO_2_.

FTIR spectra of prepared ZrO_2_ and Pt(1%)-ZrO_2_ are shown in Figure 7. Peaks at 3446 and 1628 cm^−1^ corresponding to the ν(O–H) and δ(OH) vibrations of H_2_O can be seen. In general, residual water and hydroxy groups are found in the materials regardless of the used preparation method [37]. The FTIR spectra of both samples show bands between 500–1190 cm^−1^, which correspond to Zr–O stretching vibrations. Two broad bands at 1380 and 1432 cm^−1^ appeared due to the carbonate group. These FTIR results are similar to others reported in literature [3–4].

### Catalytic activity

**Figure 8:**
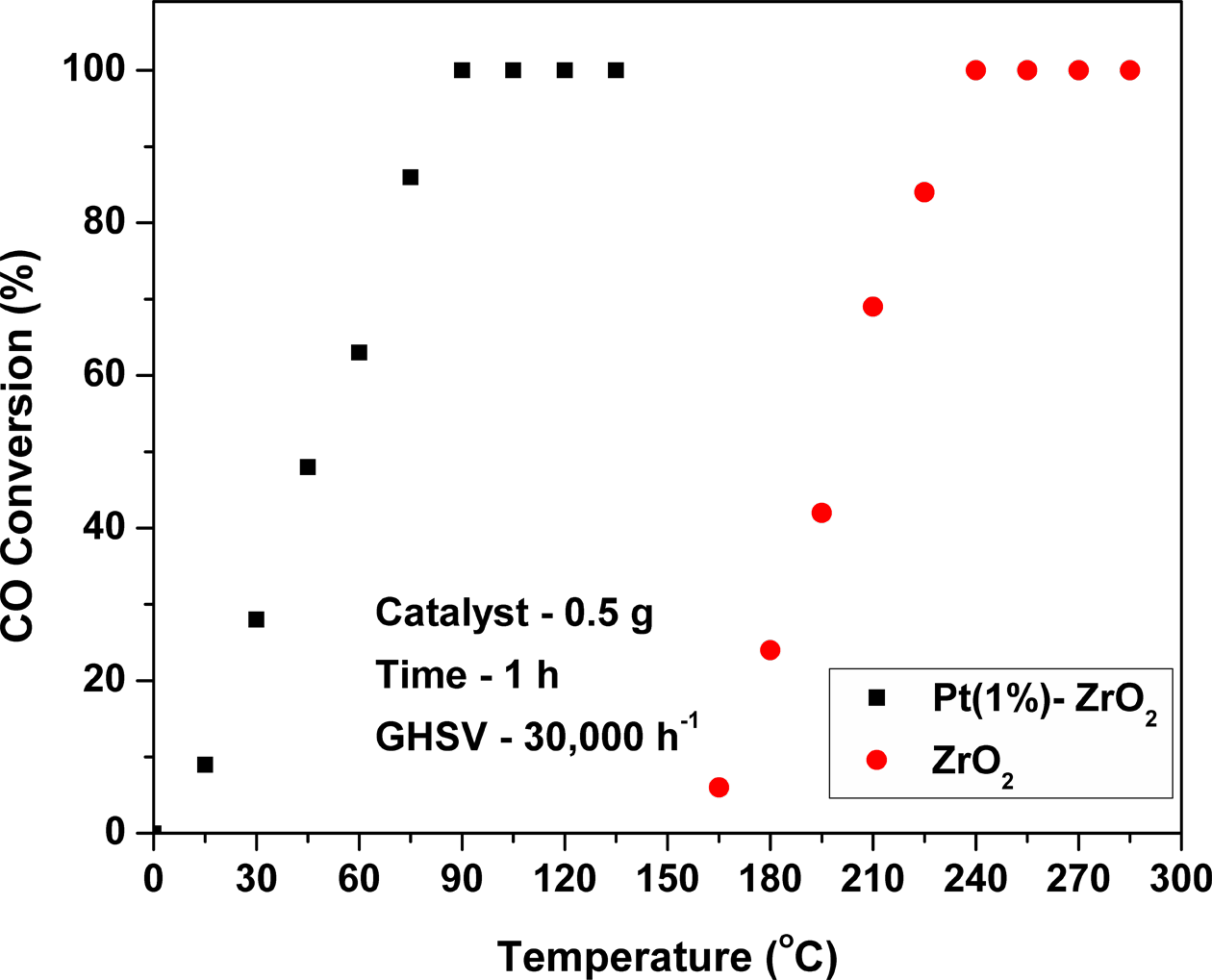
CO conversion as a function of the temperature for Pt(1%)-ZrO_2_ and ZrO_2_ (catalyst: 0.5 g, CO: 500 ppm, O_2_: 20% Ar balance, GHSV: 30,000 h^−1^).

The catalytic activity of ZrO_2_ and Pt(1%)-ZrO_2_ was evaluated for the oxidation of CO. The CO conversion was measured at different temperatures as shown in Figure 8. At 150 °C, there was no CO conversion obtained for ZrO_2_. With increase in temperature, the CO conversion also increases. The CO conversion with pure ZrO_2_ started at 175 °C with a conversion rate of about 1%. With the Pt(1%)-ZrO_2_ catalyst, the conversion began at 15 °C with a conversion rate of 2%. At a temperature of 90 °C (*T*_100_, the temperature at which 100% conversion is achieved), 100% conversion was achieved for Pt(1%)-ZrO_2_, whereas in case of ZrO_2_ this conversion rate was obtained only at a temperature of 240 °C. These results show a significantly higher catalytic activity for Pt(1%)-ZrO_2_ compared to ZrO_2_. It is because of synergic effect between Pt and Zr components, which results in an increase of the oxygen vacancies and the oxygen mobility and also improves the thermal stability.

From the XRD results, it was suggested that addition of Pt into ZrO_2_ lattice created lattice distortions, which results in oxygen vacancies. From these results, it is suggested that oxygen vacancies, high specific surface area, and the small particle size influence the catalytic activity for CO oxidation.

The catalytic activity of bare ZrO_2_ prepared by solution combustion for CO oxidation reaction is the highest in comparison to reported ZrO_2_ and other supports in literature [37–40]. The *T*_100_ value of ZrO_2_ is 240 °C, whereas much higher temperatures are reported for other catalysts [38–43]. Thus, solution combustion provides not only highly active but also stable catalysts for the oxidation of CO.

### Stability of Pt(1%)-ZrO_2_

**Figure 9:**
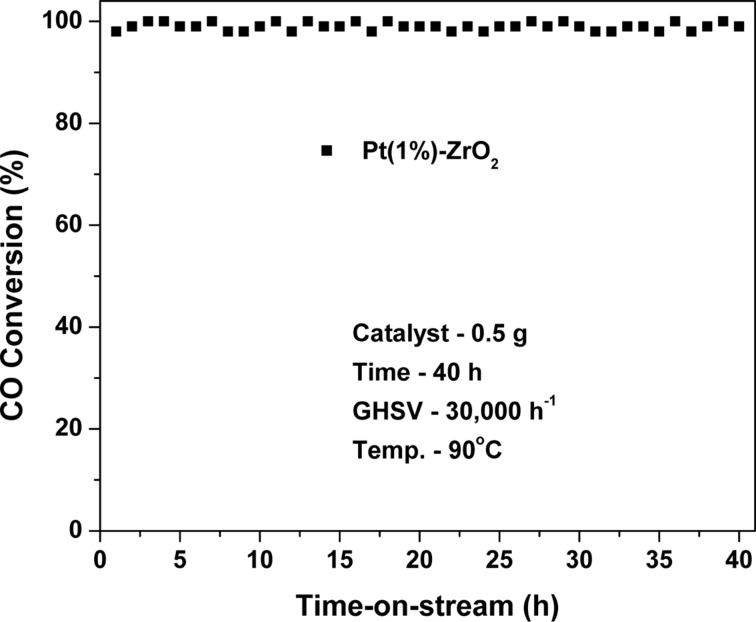
Time-on-stream stability test of Pt(1%)-ZrO_2_ for CO conversion (catalyst: 0.5 g, CO: 500 ppm, O_2_: 20% Ar balance, GHSV: 30,000 h^−1^, *T* = 90 °C).

The time-on-stream stability test was performed with Pt(1%)-ZrO_2_ over 40 h as shown in Figure 9. For this purpose, 0.5 g of catalyst was inserted into a vertically fixed quartz reactor, which was maintained at temperature of 90 °C. The stability test results show a constant CO conversion during the test, which indicates the excellent stability of Pt(1%)-ZrO_2_. The obtained results are due to improvement in thermal stability of the catalyst by the introduction of the noble metal.

### Effect of reaction conditions on CO oxidation over Pt(1%)-ZrO_2_

**Figure 10:**
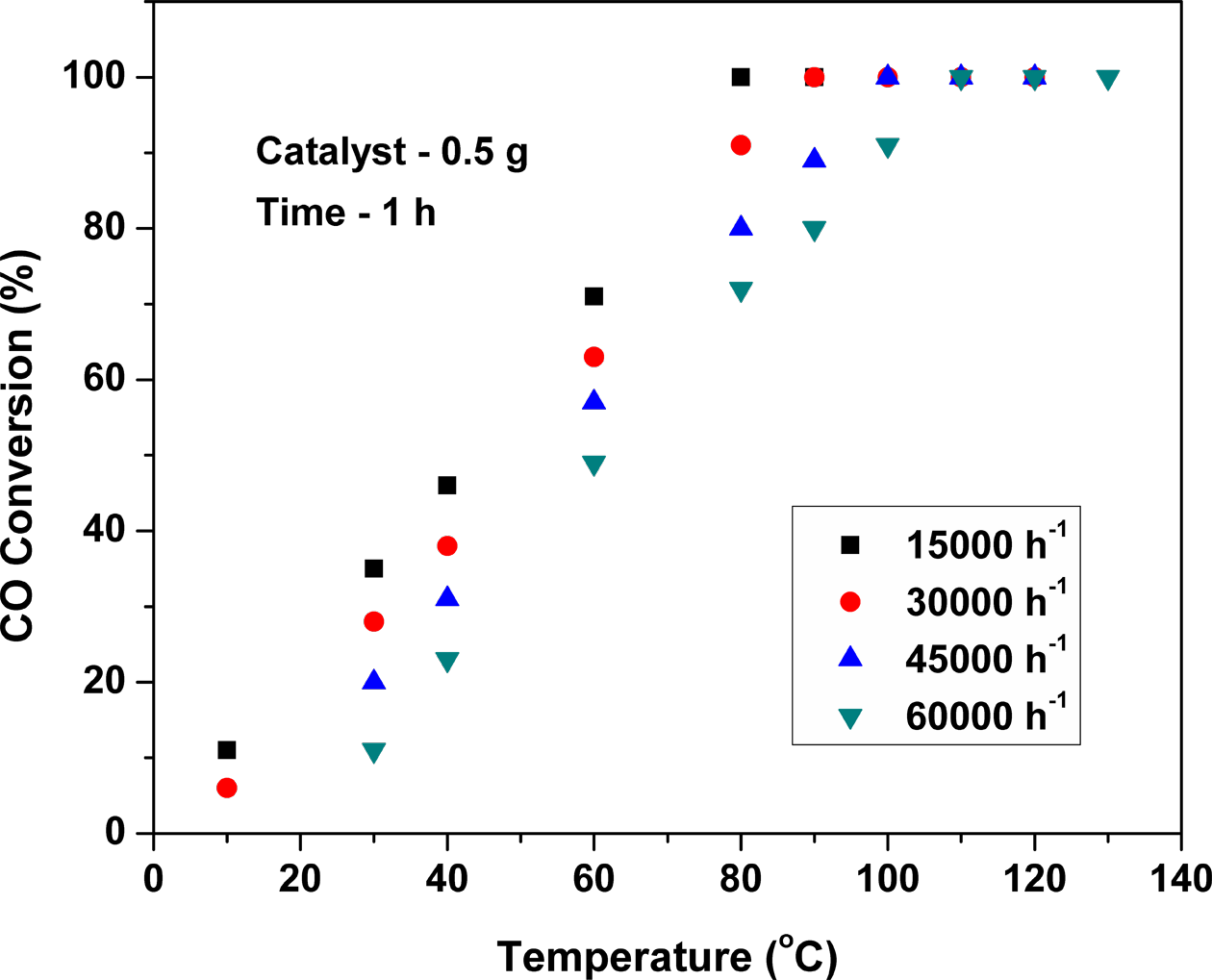
Effect of GHSV on CO conversion over Pt(1%)-ZrO_2_.

**Figure 11:**
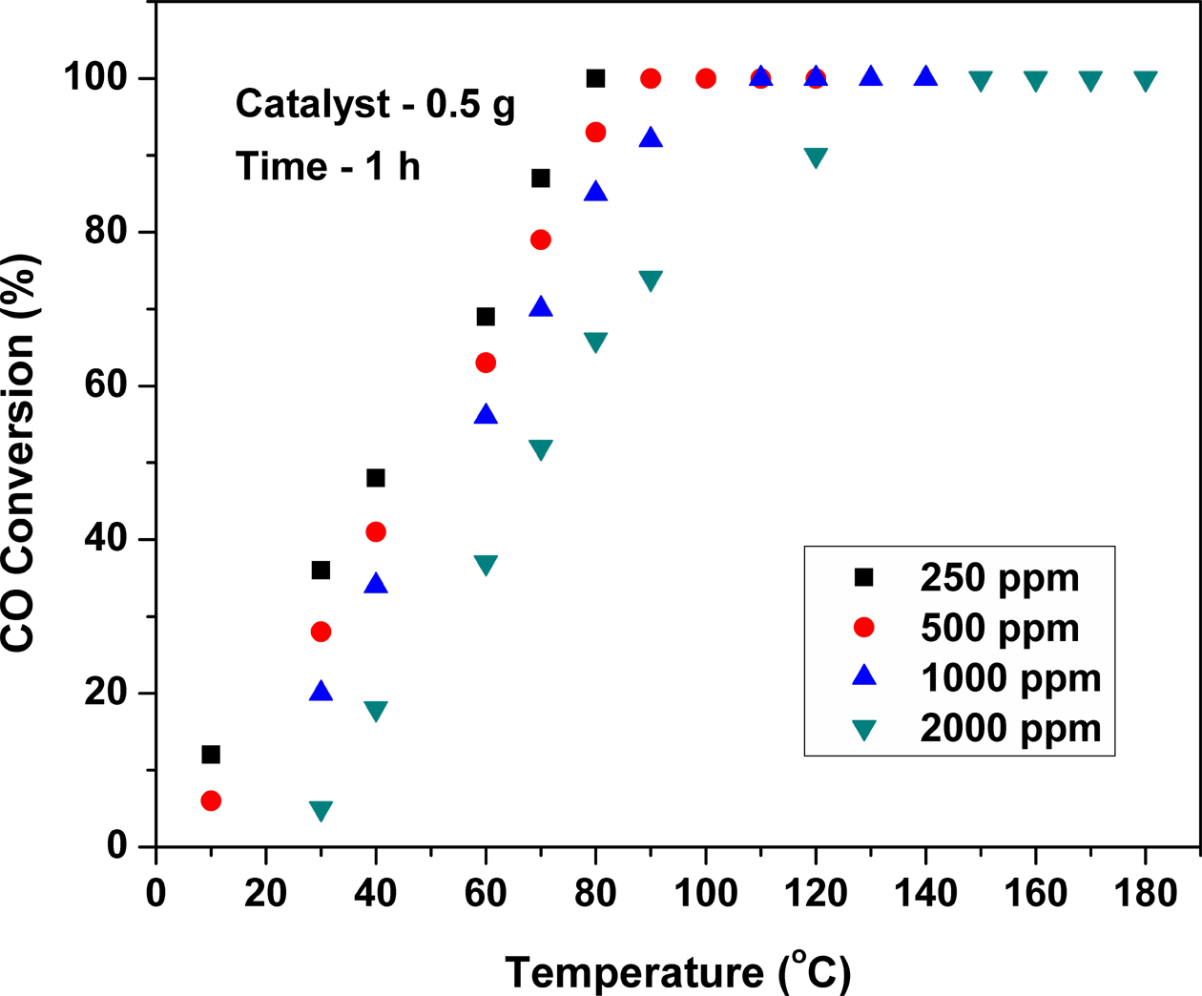
Influence of the initial CO concentration on the CO conversion over Pt(1%)-ZrO_2_.

Figure 10 and Figure 11 show the influence of the gas hourly space velocity (GHSV) and initial CO concentration on the CO oxidation over Pt(1%)-ZrO_2_. With increasing the GHSV from 15000 h^−1^ to 60000 h^−1^, the CO conversion decreases gradually. However, the obtained *T*_100_ conversion even at the maximum tested GHSV of 60000 h^−1^ is below 140 °C. For the measurement with different initial CO concentrations, the GHSV was fixed at 30000 h^−1^. The CO conversion decreases slightly, when the initial CO concentration is changed from 250 to 1000 ppm. A larger decrease in CO conversion was observed when the initial CO concentration was increased from 1000 to 2000 ppm.

## Conclusion

Nanocrystalline ZrO_2_ was successfully prepared by solution combustion, which is simple and capable of producing high-purity materials. TGA showed that the prepared ZrO_2_ nanoparticles are thermally stable up to 700 °C. XRD and TEM studies confirmed that ZrO_2_ particles formed were in the size range of nanometers.

The prepared ZrO_2_ nanoparticles showed good catalytic activity for CO oxidation at a high space velocity of 30,000 h^−1^ and achieved 100% CO conversion at 240 °C. This is the highest conversion rate reported in the literature for bare ZrO_2_. The ZrO_2_ catalyst doped with 1% Pt was prepared to demonstrate the potential of ZrO_2_ as a catalyst support. This supported Pt catalyst was also scrutinized for CO oxidation and experimental results showed 100% CO conversion at 90 °C, which is a very low temperature compared to bare ZrO_2_. Pt(1%)-ZrO_2_ also showed excellent stability during CO oxidation. Through solution combustion, Pt was introduced into ZrO_2_ lattice yields various different synergic effects, such as enhancement of oxygen mobility and increase of oxygen vacancies. This incorporation of Pt also improves the thermal stability of the catalyst. The effect of GHSV and initial CO concentration showed that with increase in GHSV and CO concentration, the CO conversion decreased.

Further work is in progress to explore the effect of different calcination temperatures of ZrO_2_ and the amount of fuel on ZrO_2_ nanoparticles and their catalytic activity for CO oxidation. In later stages, it is also planned to work with different Pt percentages on ZrO_2_ to further lower down the temperatures required for CO oxidation.

## Experimental

### Materials synthesis

All the used chemicals for the synthesis purpose were used as such with no further purification. Zirconyl nitrate hexahydrate (Fischer Scientific), hexachloroplatinic acid hexahydrate (Alfa Aesar), and urea (Merck) were used as Zr, Pt precursors and fuel. The ZrO_2_ nanoparticles were prepared by solution combustion. For the synthesis of ZrO_2_ nanoparticles, a small amount of deionized water (10–15 mL) was added to a mixture zirconyl nitrate and urea. The ratio of zirconyl nitrate and urea was maintained at 5:1. The mixture of zirconyl nitrate, urea and deionized water was stirred vigorously which resulted into a clear solution. This clear solution was inserted into the furnace and the temperature was maintained at 400 °C. The water evaporated and the reactant mixture ignited resulting in a solid material. The obtained product was then ground into fine powder and calcined at 500 °C for 4 h. Similarly, 1% Pt was doped into ZrO_2_ by solution combustion. For the synthesis of Pt-ZrO_2_ catalyst, the fuel to metal molar ratio [urea/(Zr+Pt)] was maintained at 5:1.

### Materials characterization

The powder XRD data of ZrO_2_ and Pt-doped ZrO_2_ were collected with the help of a Rigaku X-ray diffractometer (DMAX IIIVC) with Ni-filtered Cu Kα radiation (λ = 1.542 Å) over a range of 2θ from 20 to 80°. The data were obtained with an step size of 0.02° and a scan rate of 0.5°/min. The specific surface area of the samples was obtained using a BET instrument using N_2_ as adsorbent (Micrometrics, ASAP 2010). UV–vis absorbance measurements were carried out on a Shimadzu spectrophotometer (model UV-1800) in the range of 200–700 nm. Thermal analysis of the prepared ZrO_2_ samples was performed using TGA thermal analyzer (STA-1500 Model) at a heating rate of 10 °C/min under ambient atmosphere. TEM micrographs were obtained on Tecnai G^2^-20 Twin (FEI) transmission electron microscope operated at 200 kV. The prepared samples were dispersed in ethanol, and after 30 min of ultrasonication they were deposited and dried on carbon-coated Cu grids. The FTIR spectra of the ZrO_2_ samples were recorded on a Nicolet iS50 FT-IR spectrometer using KBr pellets in the range 500–4000 cm^−1^.

### Catalytic activity

The CO oxidation reaction was performed in a quartz reactor (inner diameter of 14 mm) under atmospheric pressure. 0.5 g of catalyst was inserted into the quartz tube. The reaction was carried out at different temperatures. For experiments, a mixture chamber was used to generate gas mixtures. The overall flow rate of the gas mixture which consists of 20% O_2_ balanced by Ar gas and 500 ppm CO was maintained at 100 mL/min. Gas chromatography (Nucon-5765) was used to analyze the effluent stream using a 5 Å molecular sieve column and a thermal conductivity detector (TCD). Each run was tested for at least 60 min to achieve a steady state. The CO conversion was measured as follows:

[1]